# Small, smooth, nonmobile cardiac myxoma detected by transesophageal echocardiography following recurrent cerebral infarction: a case report

**DOI:** 10.1186/s13256-017-1298-z

**Published:** 2017-05-10

**Authors:** Yuki Saito, Yoshihiro Aizawa, Koyuru Monno, Koichi Nagashima, Sayaka Kurokawa, Shunji Osaka, Takayoshi Akimoto, Satoshi Kamei, Masashi Tanaka, Atsushi Hirayama

**Affiliations:** 1 Division of Cardiology, Department of Medicine, Nihon University School of Medicine, 30-1 Ohyaguchi-kamicho, Itabashi-ku, Tokyo, 173-8610 Japan; 2 Department of Cardiovascular Surgery, Nihon University School of Medicine, Tokyo, Japan; 3 Division of Neurology, Department of Medicine, Nihon University School of Medicine, Tokyo, Japan

**Keywords:** Cardiac tumor, Cerebral infarction, Echocardiography

## Abstract

**Background:**

Cardiac myxoma is known to cause repeated events of cerebral embolism. Soft and irregularly shaped myxomas with high mobility are associated with a higher occurrence of cerebral embolism. In contrast, nonmobile cardiac myxomas with a round regular shape are rarely considered to be a cause of cerebral embolism. In this case, we present a patient with recurrent cerebral embolism associated with a small and nonmobile cardiac myxoma of round regular shape.

**Case presentation:**

A 76-year-old Japanese man presented to our hospital with weakness in his right upper extremity. He had a history of right frontal lobe infarction in the previous month. T2-weighted magnetic resonance imaging revealed an area of hyperintensity in the left precentral gyrus, indicating acute cerebral infarction. Transthoracic echocardiography revealed normal left ventricular function and no abnormalities. However, transesophageal echocardiography showed a small and nonmobile left atrial tumor with round regular shape attached to the ostium secundum of the atrial septum. Based on these findings, we diagnosed recurrent cerebral infarction due to embolization caused by left atrial myxoma, and cardiac tumor extraction was performed on hospitalization day 36. The excised tumor measured 0.6 × 0.6 × 0.5 cm and was diagnosed as cardiac myxoma by histologic examination.

**Conclusions:**

Even small and nonmobile cardiac myxomas with a round regular shape may cause recurrent cerebral infarction. The diagnosis of this type of atrial myxoma is elusive and transesophageal echocardiography was an effective method of detection. In a clinical situation, this type of cardiac myxoma may be overlooked as a cause of cerebral infarction.

## Background

Cardiac myxoma is the most common type of primary intracardiac tumor in adults, and is histologically benign. This tumor is most often located in the left atrium (LA) [1]. The reported diameter of cardiac myxomas ranges from 2 to 6 cm [2–4]. Cardiac myxoma is known to cause repeated cerebral embolisms [5]. Soft and irregularly shaped myxomas with high mobility have been associated with a higher occurrence of cerebral embolism [2, 3].

In contrast, reports of nonmobile cardiac myxomas with a round regular shape that cause cerebral embolism are rare [6]. Although there have been many case reports and series about myxoma and secondary cerebral infarction, almost all of the cardiac myxomas causing recurrent cerebral infarction demonstrated a large irregular shape with a mobile component [2–4, 6–9]. In this report, we present a case of recurrent cerebral embolism associated with a small and nonmobile cardiac myxoma of round regular shape. This type of cardiac myxoma can be elusive, and transesophageal echocardiography (TEE) was an effective means to detect it.

## Case presentation

**Fig. 1:**
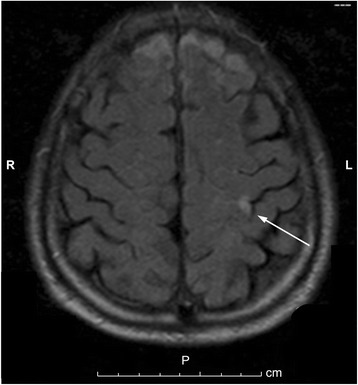
T2-weighted brain magnetic resonance image, showing an area of hyperintensity in the left precentral gyrus (*arrow*)

**Fig. 2:**
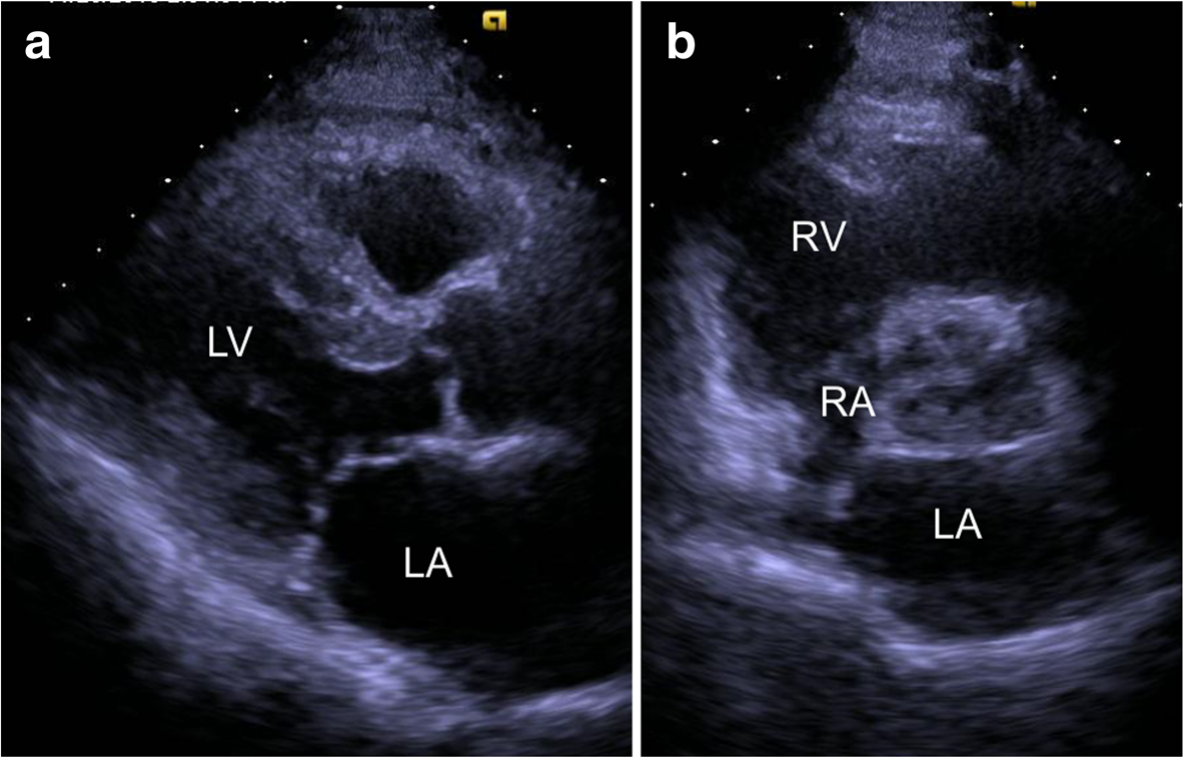
Long-axis (**a**) and modified short-axis (**b**) transthoracic echocardiograms before surgical resection, showing no left atrial mass. Abbreviations: *LA* left atrium, *LV* left ventricle, *RA* right atrium, *RV* right ventricle

**Fig. 3:**
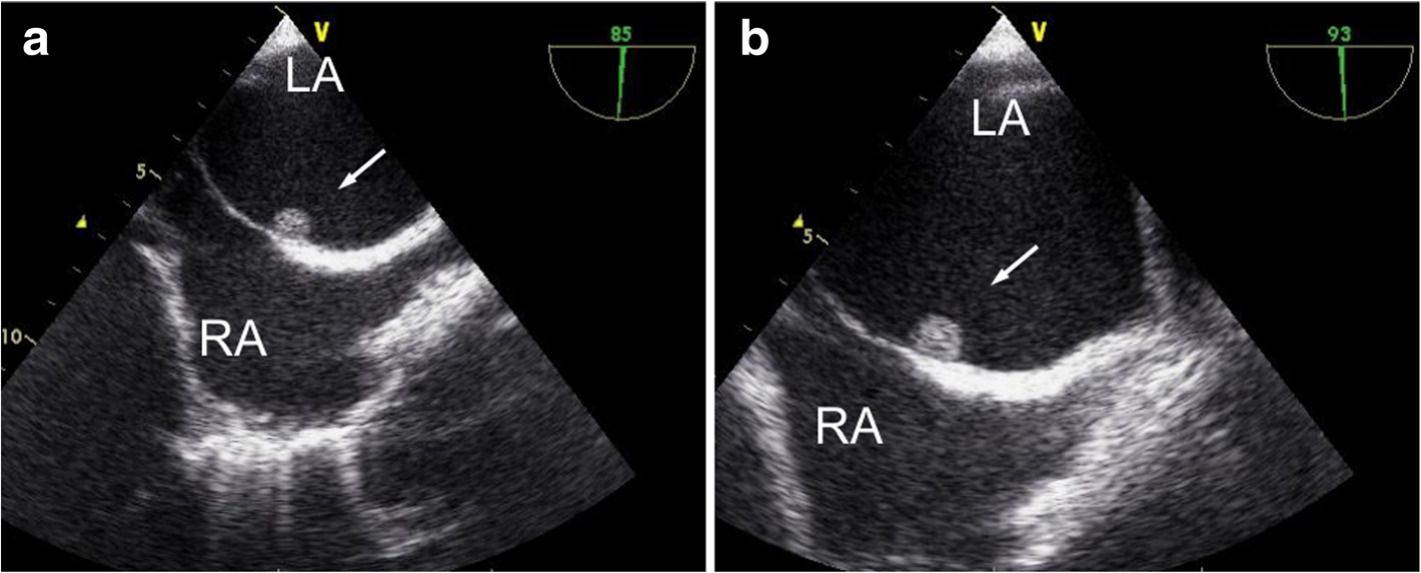
**a**, **b** Bicaval transesophageal echocardiograms, showing an acaulescent immobile mass in the left atrium arising from the atrial septum (*arrow*). Abbreviations: *LA* left atrium, *RA* right atrium

A 76-year-old Japanese man presented to our hospital with weakness in his right upper extremity. He had a history of right frontal lobe infarction in the previous month, as well as diabetes, hypertension, and abnormal lipid metabolism. On admission, he was conscious. His body temperature was 36.3 °C, blood pressure was 155/74 mmHg, pulse rate was 60 beats/minute, and oxygen saturation was 98% (ambient air). Cardiovascular, respiratory, and abdominal examinations were unremarkable, while detailed neurologic assessment revealed weakness in his right hand and a positive Barré test of his right upper extremity. Laboratory findings were within accepted reference ranges. His inflammatory markers, such as white blood cell and C-reactive protein, were also in the normal range. Electrocardiography (ECG) showed a normal sinus rhythm without ST-T segment change. Chest radiography revealed a cardiothoracic ratio of 48%. Brain magnetic resonance imaging showed an area of hyperintensity in the left precentral gyrus, indicating acute cerebral infarction, consistent with his weakness in the right upper extremity (Fig. 1). Magnetic resonance angiography of his left cervical internal carotid artery showed no abnormal findings. Transthoracic electrocardiography (TTE) revealed normal left ventricular function and no abnormalities except for LA dilation (Fig. 2a, b). Altogether, the ECG had a normal sinus rhythm, our patient had no history of atrial fibrillation, and the TTE indicated no mass in his heart; however, cerebral infarction was recurrent and occurred in the cortex (left precentral gyrus) without cervical arterial stenosis. Therefore, we suspected cardiac embolism as the cause of cerebral infarction and performed TEE. TEE showed a small LA tumor attached to the ostium secundum of the atrial septum (Fig. 3a, b).

**Fig. 4:**
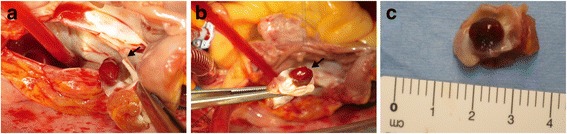
Intraoperative findings. **a**, **b** The tumor was attached to the atrial septum (*arrows*). **c** On macroscopic examination, the excised tumor was reddish brown with a smooth surface

He was diagnosed as having cerebral infarction, and was initially treated with edaravone and argatroban. Because the cardiac tumor detected by TEE was considered to be the cause of cerebral infarction, on hospital day 36, complete tumor resection, followed by atrial septal defect repair using an equine pericardial patch, was performed (Fig. 4a, b). The surgery was performed via median sternotomy under cardiopulmonary bypass with biatrial cannulation. On macroscopic examination, the excised tumor was 0.6 × 0.6 × 0.5 cm, reddish brown, and smooth (Fig. 4c). Histologic examination showed the presence of myxoid stroma containing polygonal or stellate cells arranged in groups and strings; these findings were consistent with cardiac myxoma. Our patient had an uncomplicated postoperative recovery, and his neurologic symptoms improved with conservative treatment. He was discharged on hospital day 58.

## Discussion

The clinical features of this case were: first, that a small nonmobile atrial myxoma with round regular shape could cause recurrent cerebral infarction; and second, that TEE was a useful method to detect this elusive type of atrial myxoma.

Primary cardiac tumors are rare, with a reported incidence of approximately 0.02% [10]. Myxomas are the most common type of primary cardiac tumor in adults, and are histologically benign. Nearly 80% of myxomas are localized in the LA, and are mostly attached by a pedicle to the fossa ovalis in the atrial septum. Approximately 15 to 20% are found in the right atrium [1]. The reported mean diameter of cardiac myxomas is 2 to 6 cm [2–4]. Although benign, atrial myxomas can cause repeated events of cerebral and peripheral embolism, intracardiac obstruction, and constitutional symptoms such as fever and weight loss [11–14].

The major serious complication of atrial myxoma is recurrent ischemic cerebral infarction [5]. It has been reported that at least 25% of patients with myxoma present with ischemic neurologic events secondary to embolism [3]. The embolic source may be tumor fragments or surface emboli [15]. The risk of systemic embolism of a myxoma is related to tumor morphology. Two types of myxoma have been reported: round type, characterized by solid, round and regular shape with nonmobile surface; and polypoid type, characterized by soft and irregular shape with mobile surface [2]. The polypoid type of tumor is associated with a higher occurrence of systemic embolism than the round type [2]. This type of tumor is fragile and prone to embolization in the arteries, possibly causing multiple emboli. In contrast, emboli associated with round myxomas are rare [6]. In addition, myxoma mobility is also related to risk of embolization [3, 16]. Myxomas with high mobility present high embolization potential. Although there have been many case reports and series about myxoma and secondary cerebral infarction, almost all of the cardiac myxomas causing recurrent cerebral infarction demonstrated a large (more than 2 cm) irregular shape and mobile component [2–4, 6–9]. In this case, we found that even a small nonmobile round type of atrial myxoma could cause recurrent cerebral infarction.

TEE was an effective tool to detect this elusive from of atrial myxoma. In this case, ECG demonstrated a normal sinus rhythm, our patient had no past history of atrial fibrillation, and there was no mass in his heart by TTE; however, cerebral infarction was recurrent and occurred in the cortex (left precentral gyrus) without cervical arterial stenosis, leading us to suspect cardiac embolism as the cause of cerebral infarction and to perform TEE. Emboli frequently lodge in the middle cerebral artery (MCA) branches. Thus, most infarctions related to left atrial myxomas present in MCA distribution [16]. Our patient presented infarctions in the precentral gyrus, which would be consistent with MCA distribution. TTE may not detect tumors measuring less than 5 mm in diameter [17], which is a possible explanation of why this type of myxoma has been overlooked as a cause of cerebral infarction. Therefore, detailed cardiac examination is necessary when cerebral infarction is recurrent or has occurred in the cortex without arterial stenosis.

The most common treatment of cardiac myxoma is surgical resection of the tumor followed by repair of the defect. In addition, surgical removal of adjacent tissue has been recommended to reduce the risk of recurrence. The long-term outcome after surgical myxoma excision is excellent [18]. However, because the recurrence rate is 1 to 3% after surgery [19], careful follow-up should be performed.

## Conclusions

In the case reported here, we determined by TEE that a small nonmobile atrial myxoma with a round regular shape caused recurrent cerebral infarction. This type of cardiac myxoma may be overlooked as a cause of cerebral infarction in the absence of severe symptoms or history of cardiac problems because routine cardiac investigations may not be performed. Therefore, it is important for clinicians to perform a detailed cardiac examination when cerebral infarction has been recurrent or has occurred in the cerebral cortex without arterial stenosis.

## Abbreviations

ECG: Electrocardiography
LA: Left atrium
MCA: Middle cerebral artery
TEE: Transesophageal echocardiography
TTE: Transthoracic electrocardiography
